# The influence of electromyographic biofeedback therapy on knee extension following anterior cruciate ligament reconstruction: a randomized controlled trial

**DOI:** 10.1186/1758-2555-4-41

**Published:** 2012-11-06

**Authors:** Franz Christanell, Christian Hoser, Reinhard Huber, Christian Fink, Hannu Luomajoki

**Affiliations:** 1 Sporttherapie Christanell, Bozen, Italy; 2 Sportsclinic Austria, Innsbruck, Austria; 3 Sporttherapie Huber, Innsbruck, Austria; 4 Zürich University of Applied Sciences, Winterthur, Switzerland

**Keywords:** Anterior cruciate ligament, Rehabilitation, Biofeedback, EMG, Knee extension

## Abstract

**Background:**

Loss of knee extension and a deficit in quadriceps strength are frequently found following anterior cruciate ligament (ACL) reconstruction. The aim of this study was to investigate whether the addition of Eletromyographic Biofeedback (EMG BFB) therapy for the vastus medialis muscle to the in the early phase of the standard rehabilitation programme could improve the range of knee extension and strength after ACL reconstruction more than a standard rehabilitation programme. The correlation between EMG measurement and passive knee extension was also investigated.

**Method:**

Sixteen patients, all of whom underwent endoscopic ACL reconstruction using patellar tendon autograft, were randomly assigned to two groups:

• Control group (8 patients): standard rehabilitation protocol; with full weight-bearing postoperative, knee brace (0° extension, 90° flexion), electrical stimulation, aquatics and proprioceptive training.

• The EMG BFB group (8 patients): EMG BFB was added to the standard rehabilitation protocol within the first postoperative week and during each session for the next 6 weeks.

Each patent attended a total of 16 outpatient physiotherapy sessions following surgery. High-Heel-Distance (HHD) Test, range of motion (ROM) and integrated EMG (iEMG) for vastus medialis were measured preoperatively, and at the 1, 2, 4 and 6-week follow ups. Additionally, knee function, swelling and pain were evaluated using standardized scoring scales.

**Results:**

At 6 weeks, passive knee extension (p < 0.002) and the HHD Test were significantly (p < 0.01) better in the EMG BFB group compared to controls. Integrated EMG (vastus medialis) of the EMG BFB group also showed a significant increase after 2 (p < 0.01) and 6 (p < 0.01) weeks. At the 6-week follow up, no significant (p > 0.01) differences were found between the two groups for the assessment of knee function, swelling and pain.

**Conclusion:**

The results indicate that EMG BFB therapy, in the early phase of rehabilitation after ACL reconstruction, is useful in enhancing knee extension. Improved innervation of the vastus medialis can play a key role in the development of postoperative knee extension. EMG BFB therapy is a simple, inexpensive and valuable adjunct to conventional therapeutic modalities.

## Background

Over the last two decades, the human anterior cruciate ligament (ACL) has been widely studied. Investigation has focused mainly on mechanical and biological properties [1]. This development has been in response to the continuous increase in ACL injuries, attributing to the increase in leisure activities, such as snowboarding or roller blading [2,3].

Extensive literature research shows that after ACL reconstruction, knee extension range deficits [4-8], decrease in quadriceps muscle strength [9-14] and anterior knee pain, [15-18] frequently create difficulties for rehabilitation. The underlying mechanism for the loss of knee extension can be summarized in preoperative [5], intraoperative [4] and postoperative [19] deficits. A loss of knee extension often leads to an extended rehabilitation period and additional surgeries [4,5]. The relationship between persistent loss of knee extension and deficits of activation for vastus medialis have been described [8]. Changes in the sensorimotor system, caused by protection from the central neuronal system, are suspected to play an important role [11,20-24]. Positive effects of Electromyographic Biofeedback (EMG BFB) training [25,26] on knee extension deficit have already been published. However, there were a number of shortcomings to these studies: no preoperative measurements were performed to allow the improvement between the preoperative phase and the end of the postoperative rehabilitation programme to be identified. No short term effects following the first 6-week postoperative phase were presented. Furthermore, no direct comparison between EMG measurements and extension deficit has been made. And, finally, the use of a preventive, early implementation of EMG-assisted training after ACL reconstruction has not yet been studied.

The aim of this study was to investigate whether Electromyographic Biofeedback (EMG BFB) therapy for the vastus medialis muscle has an add-on effect to the standard rehabilitation programme in improving knee extension range and strength in the early phase of rehabilitation after ACL reconstruction. The correlation between EMG measurement and passive knee extension was also investigated.

## Method

### Participants

Sixteen patients were recruited from the University Clinic of Innsbruck (Austria), of which 12 were male and 4 female. They had a mean age of 30 years (range: 20–49 years). All participants underwent endoscopic ACL reconstruction by the same surgeon and the same surgical protocol using patellar tendon autograft.

### Randomisation

Between March and October, 2005, consecutive patients of the orthopaedic surgeon conducting the operations were included in the study. The inclusion criteria included free range of motion, pain free and without inflammation, isolated ACL rupture and cartilage damage < level 2.

The patients were enrolled for therapy by telephone through the secretary of the surgeon. The recruited patients were chronologically randomized into one of two treatment groups; with EMG BFB (n = 8), or, without EMG BFB (n = 8). The treating therapist had no knowledge of the group to which a patient was allocated, and the patients were previously unknown to him. After randomisation the therapist knew who gets BFB-therapy and who not.

### Intervention

The Control group (8 patients) received a standard rehabilitation protocol with full postoperative weight-bearing, knee brace (0°extension, 90° flexion), electrical stimulation (without Biofeedback-intervention but combined with maximal voluntary isometric knee extension), aquatics and proprioceptive training. For the EMG BFB group (8 patients), EMG BFB (Myotrainer© - Insight Instruments - Austria) was added within the first week and during each session for the first 6 weeks. Biofeedback therapy contained exercises for optimal vastus medialis activation under real-time visual and acoustic control on a laptop. Exercises included maximum isometric knee extension, one-leg standing, mini squats, etc. During each session, the patient should enhance his own maximum threshold EMG value. Each patient attended 16 outpatient physiotherapy sessions following surgery, which were all supervised by the same therapist (Table 1).

**Table 1:** Division key of sessions: 16 Physiotherapy (PT), 8 Electrostimulation (ES), 8 Manual Lymphatic drainage (MLD), 8 Underwater Hydrotherapy (UWT)

**1st week**	**2nd week**	**3rd week**	**4th week**	**5th week**	**6th week**
3 × PT	3 × PT	3 × PT	3 × PT	2 × PT	2 × PT
3 × ES	3 × ES	2 × ES			
3 × MLD	3 × MLD	2 × MLD			
		2 × UWT	2 × UWT	2 × UWT	2 × UWT

### Outcome measurements

High-Heel-Distance (HHD) Test, range of motion (ROM) and integrated EMG (iEMG) for vastus medialis were measured 1 week before surgery and at the 1,2,4 and 6-week post-operative follow-ups. Integrated Electromyography was used to measure muscle activation across surface electrodes. It is the common method used in scientific studies for muscle activation measurement [27]. Additionally, knee function, swelling and pain were evaluated using standardized scoring scales. A standardised questionnaire was utilized to evaluate socio-demographic parameters, sport activity and irritability, according to the guidelines of the International Knee Documentation Committee (IKDC) [24,28]. Ethical approval was given by the hospital ethical board and all patients signed a written informed consent form before entering the study.

### Measuring the knee extension and tension of vastus medialis

**Figure 1:**
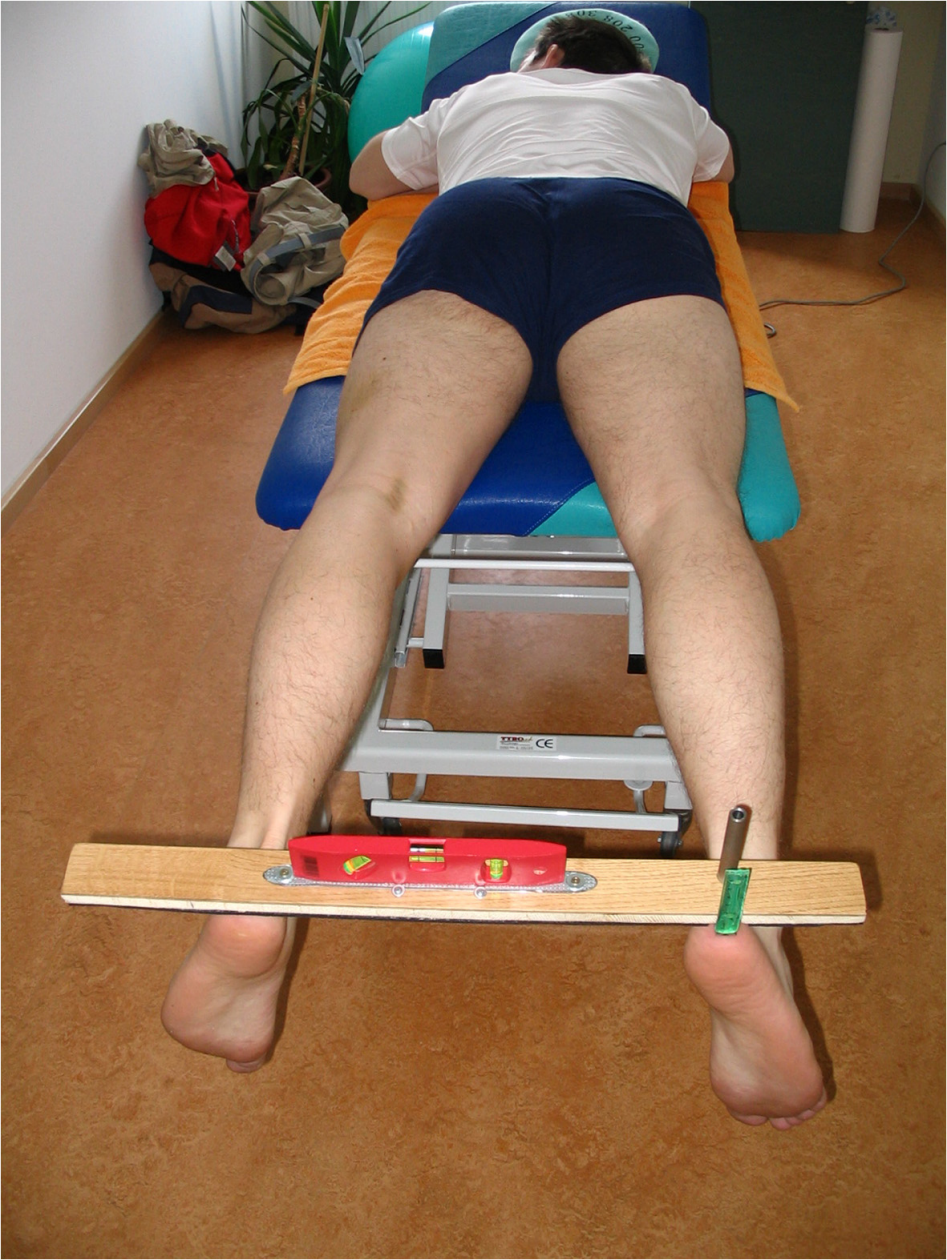
HHD Test for measuring passive knee extension.

**Figure 2:**
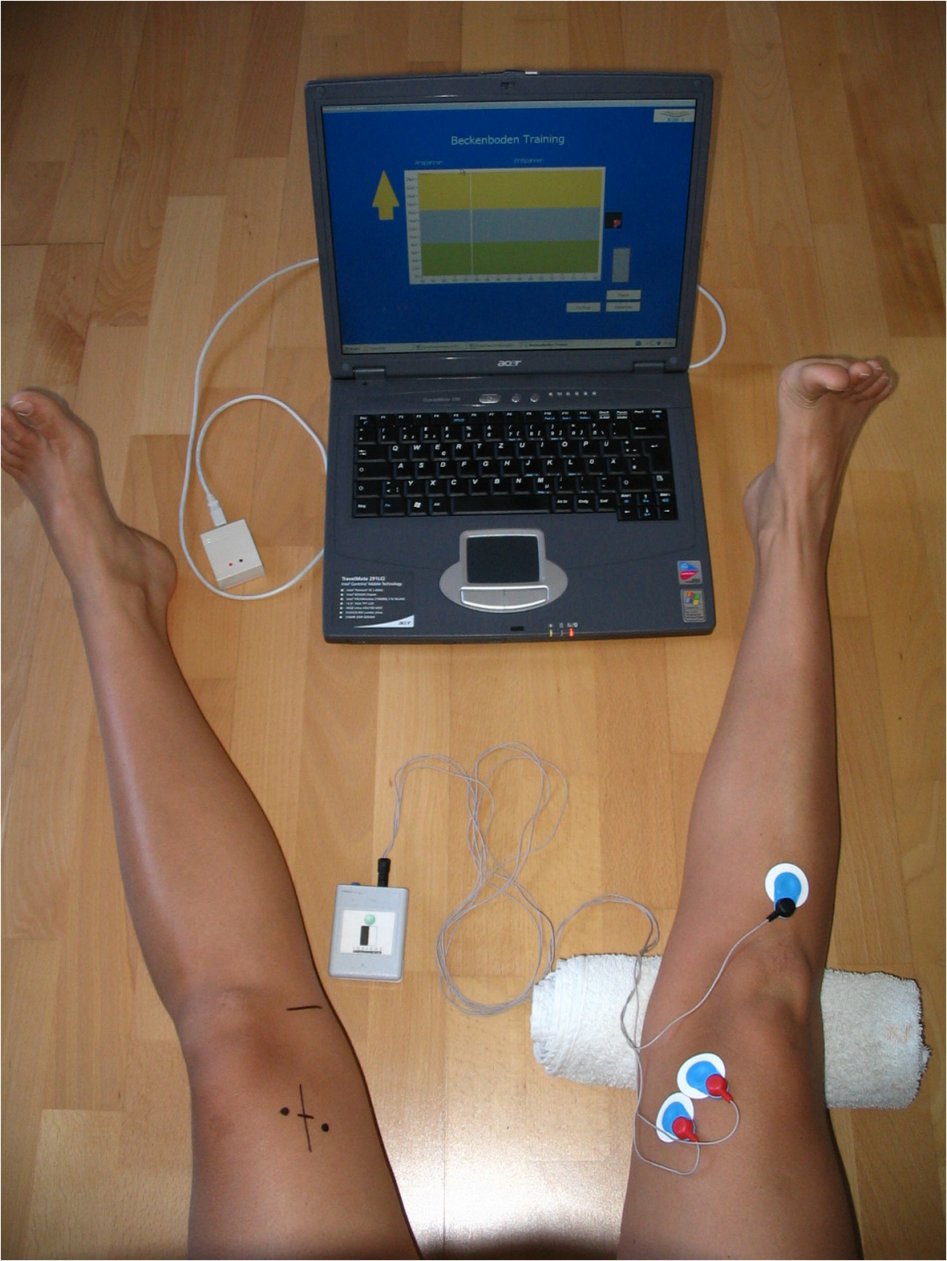
The iEMG measuring.

Figure 1 shows the application of the HHD Test which was used, alongside a goniometer, for measuring passive knee extension. The HHD Test is a simple and valuable test for evaluation of passive knee extension [29]. In a prone position (with the base of the patella over the couch) the horizontal level of the heels indicated the high-heel-difference (HHD) in millimetres. After 60 seconds the discrepancy of the two heels was measured. The integrated EMG measurement (Figure 2) was performed with the patients sitting with the knee fully extended. Skin preparation (shaving, alcohol, sand paper) and electrode positioning were used, based on standard methods by SENIAM (Surface EMG for Non-Invasive Assessment of Muscles) [13]. Round Ag/AgCL-one-way silver electrodes (Blue Sensor – Medicotest – Denmark) were used [13,27].

For a better test-retest reliability, the positions of the electrodes were calculated as the following: 20% of the distance between the medial knee joint gap and ASIS (anterior superior iliac spine) [30]. In addition to this imagined line, the electrode position was turned 45° laterally and stuck above the vastus medialis at a distance of 20 mm. The reference electrode was positioned on the tibial tuberosity. Adhesive tape was used to attach the cable onto the patient’s skin. The integrated EMG measurement (Soft- and Hardware by INSIGHT INSTRUMENTS – Austria) was carried out as follows: the baseline test and later tests followed the same procedure: the patient was required to isometrically extend their knee to maximum extension for 4 seconds, then rest for 10 seconds, the procedures were repeated for 3 times. During the 4 seconds of maximum isometric knee extension, mean values of EMG raw signal (wide pass filter 25–1000 Hz) were measured. The best mean value (Microvolt) of the three trials was taken for statistical calculations.

Within 1 week after preoperative testing, all 16 patients obtained an ACL reconstruction operation by an experienced surgeon from the Medical University of Innsbruck (Austria). All patients wore thrombosis stockings and a knee brace (0° extension, 90° flexion) following 6 weeks after operation. Ambulant physiotherapy started after the first postoperative week with the first retest. The same tests were performed after 2, 4 and 6 weeks.

### The rehabilitation protocol

The rehabilitation protocol following surgery comprised a total of 16 outpatient physiotherapy sessions of 40 minutes, 8 electrical stimulations (ES) of 20 minutes, 8 manual lymphatic drainages (MLD) of 30 minutes and 8 aquatic underwater therapies (UWT) of 30 minutes, supervised by the same therapist. Table 1 shows all sessions divided between the first 6 weeks after ACL reconstruction.

**Figure 3:**
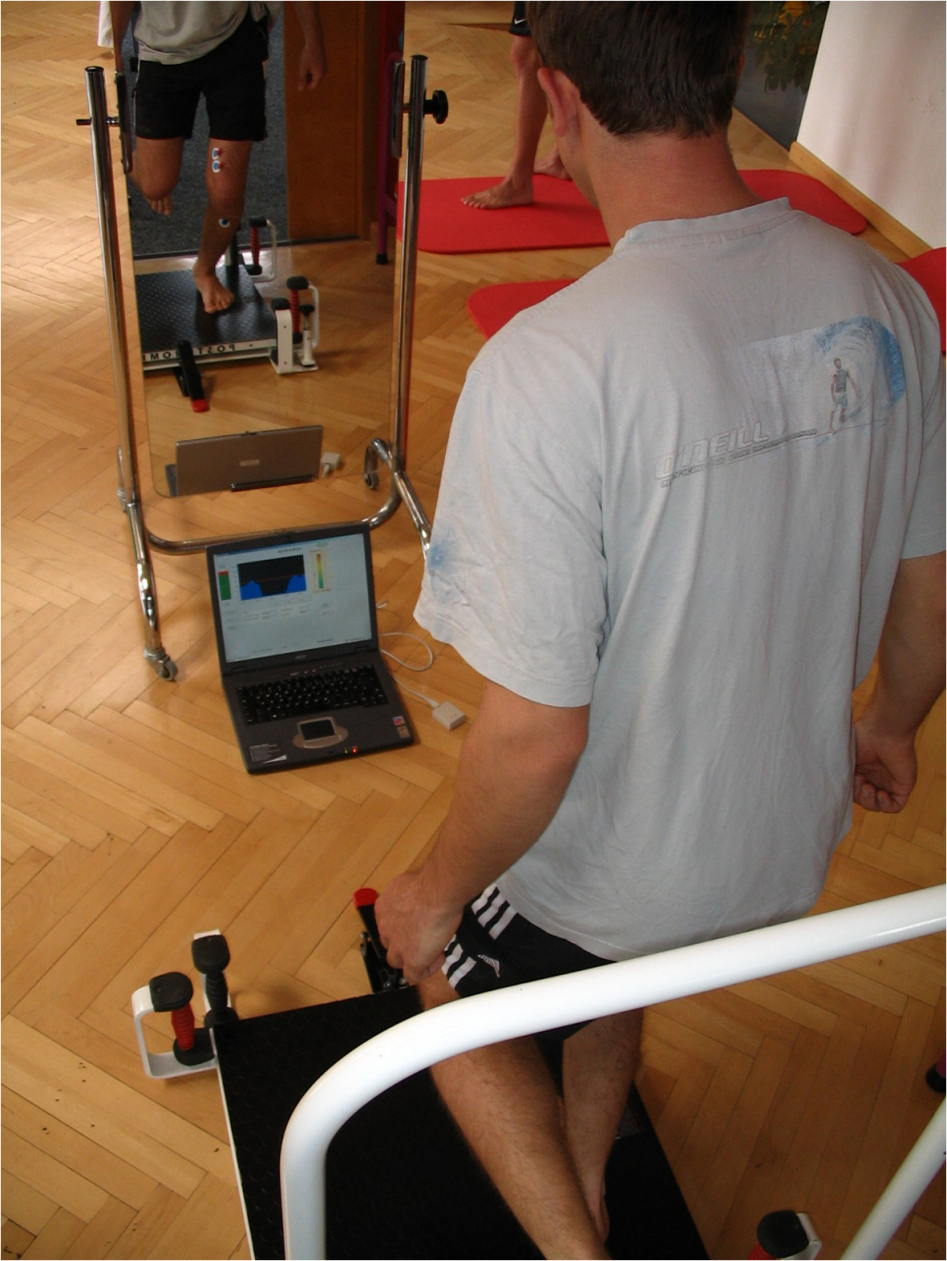
Vastus medialis-activation with BFB.

**Figure 4:**
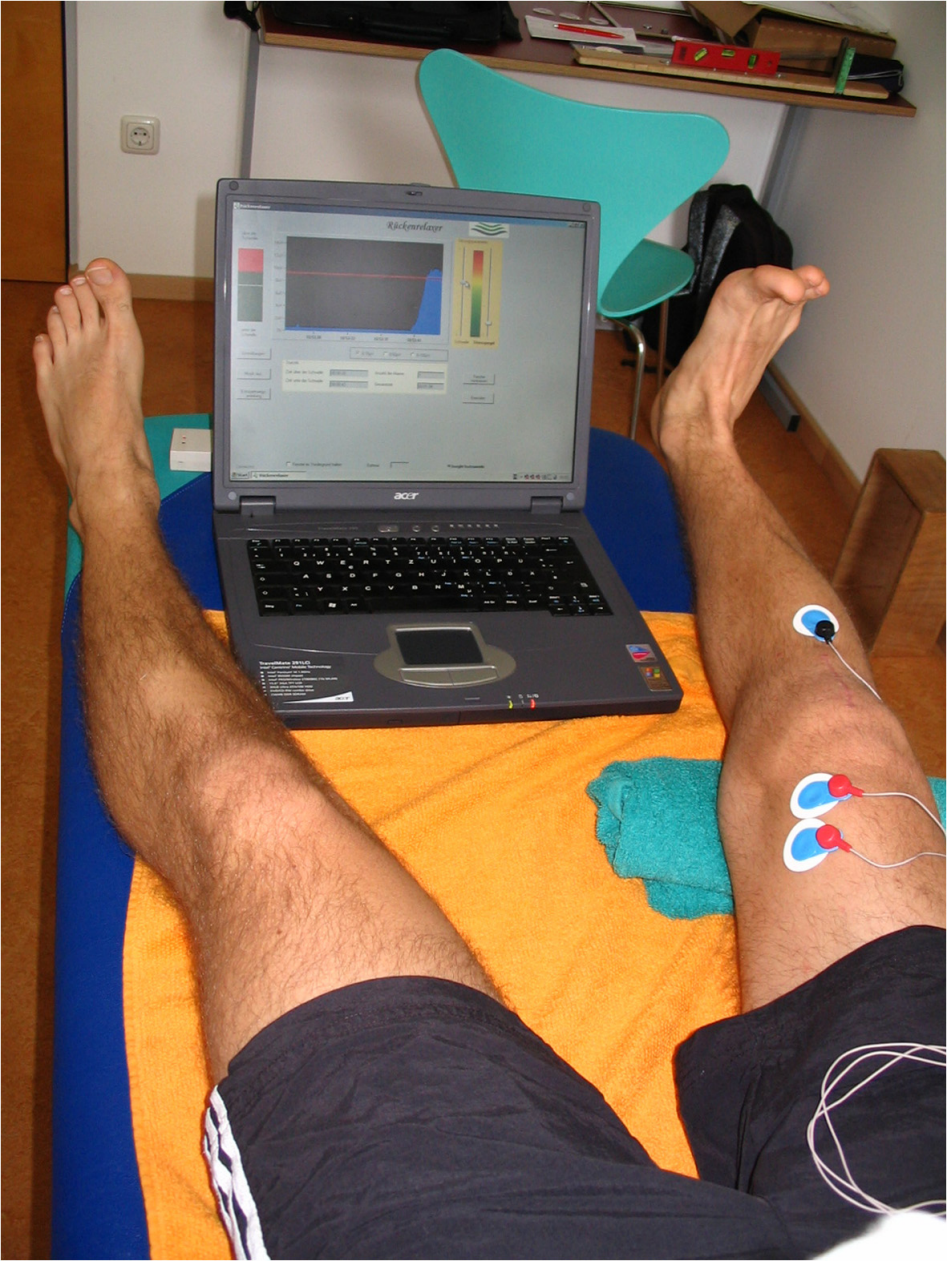
One-leg-stabilisation exercises with BFB-control on Posturomed©.

After surgery, both groups were treated with the standard protocol by the same therapist. The EMG BFB group were additionally treated with EMG BFB therapy within the first therapy session and during each session for the next 6 weeks. Visual and acoustic feedback supported optimal vastus medialis activation. Figure 3 and Figure 4 show some exercises combined with EMG BFB therapy during the physiotherapy sessions.

### Statistical methods

All data were summarised with standard descriptive statistics (mean value ± SD). Differences between the groups were calculated using Student’s t-test for parametric and Mann–Whitney-U-test for nonparametric variables. Correlations were calculated using Pearson’s coefficient of correlation test. For calculating interaction effects between the two groups, a two-way repeated measures ANOVA was used including Bonferroni adjustment; with reference to the Bonferroni adjustment, p-value less than 0.01 was considered significant. All p-values were two-tailed. The statistical analysis was performed with version 12.0 of SPSS for Windows; the graphics were created with Microsoft EXCEL (version XP 2002 for Windows).

## Results

All patients attended all the therapy sessions. There were no drop-outs or adverse events. The amount of therapy was identical for both groups, except that the EMG BFB group combined manual therapy and exercises with acoustic and visual Biofeedback for vastus medialis in the first week.

Table 2 displays the socio-demographic data of the patients. Almost all (98.3%) of the patients reported that the ACL injury had occurred during a sporting activity, mostly skiing (50%) and soccer (25%). Concerning the frequency of sporting activity, 56% fell into the category, “Two times per week, one hour sport “. The most frequent time period between ACL injury and reconstruction was 6–12 weeks in both groups (BFB group 25% and Control group 31%). Most of the patients (62.5%) received preoperative physical therapy. Neither preoperative physical therapy nor time period showed significant differences between the two groups.

**Table 2:** Comparison of social demographic parameters (mean value ± standard deviation) between the two groups; *Denotes a significant difference at p < 0.05

	**EMG BFB group**	**Control group**	**p**-value(*)
**age** (**years**)	32.9 (±9.3)	27.1 (±6.2)	0.166
**height** (**cm**)	180.3 (±8.7)	176.1 (±11.9)	0.442
**weight** (**kg**)	83.9 (±16.9)	71.9 (±11.8)	0.122
**BMI** (**kg**/**m**^**2**^)	25.6 (±3.3)	23.5 (±3.4)	0.225

There were no significant differences between the two groups regarding the number of therapy session received (Table 3).

**Table 3:** Number of therapy sessions (mean value ± SD) during 6 weeks of rehabilitation; PT = physiotherapy, ES = electrical stimulation, MLD = lymphatic drainage, UWT = aquatics)

	**PT**	**ES**	**MLD**	**UWT**
**EMG BFB group**	16 (± 0)	8 (± 0)	9 (± 1)	7.5 (± 0.9)
**Control group**	16 (± 0)	8 (± 0)	8.5 (± 0.9)	7.4 (± 0.9)

### Range of motion (ROM)

The results of ROM reported on mean values with SD of passive motions in the sagittal plane. Passive flexion and extension (with goniometer) and HHD Test were measured 1 week before surgery and at the 1,2,4 and 6-week post-operative follow-ups comparing the involved and non-involved leg.

**Figure 5:**
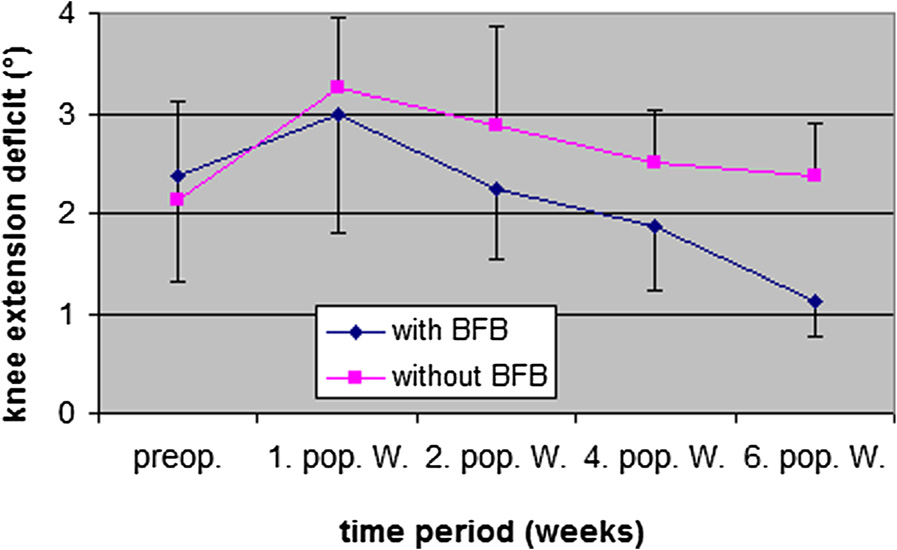
Knee extension deficit (°) (mean value ± SD) between involved and non-involved knee pre- and postoperative 6 weeks follow up (4 = 11-15°, 3 = 6-10°, 2 = 3-5°, 1 = <3° / preop. = preoperative, 1., 2., 4., and 6. pop. W. = 1., 2., 4., and 6. postoperative week).

Concerning passive knee extension deficit (difference in degrees between involved and non-involved leg), both groups were preoperatively similar (BFB group 2.4 ± 1.0 = 6.5°/ Control group 2.1 ± 1.0 = 5.1°) (Figure 5). 62% of all patients had a preoperative extension deficit in the contra lateral comparison of <5°. After 1 week the BFB group improved their knee extension more than the Control group, so that after 6 weeks the BFB group (1.1 ± 0.4 = 1.4°) had an improvement of 4.9°, compared to the Control group (2.4 ± 0.5 = 6.3°). The preoperative level was lower in the BFB group (−4.1°) than in the Control group (+1.2°). The difference between the BFB group and the Control group was significant after 6 weeks (p < 0.002).

**Figure 6:**
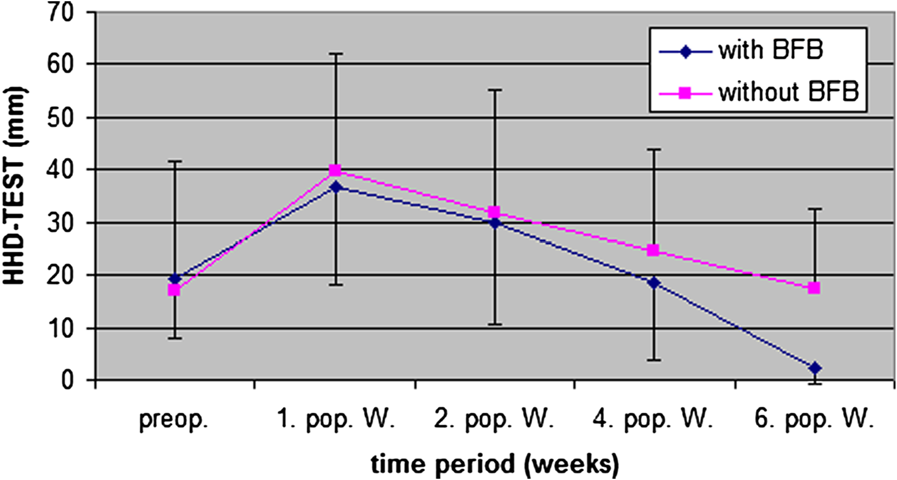
HHD Test (mm) (mean value ± SD) between involved and non-involved knee pre- and postoperative 6 weeks follow up (preop. = preoperative, 1., 2., 4., and 6. pop. W. = 1., 2., 4., and 6. postoperative week).

The results of the HHD Test (mm) showed preoperatively nearly the same mean values with SD between both groups (BFB group 19.1 mm ± 11/ Control group 16.9 mm ± 24.9) (Figure 6). During the 6 weeks rehabilitation, the BFB group improved their HHD values more than the Control group, so that after 6 weeks the BFB group (2.3 mm ± 3.2) had improved significantly (p < 0.01) more than the controls (17.4 mm ± 15.3). Compared to preoperative levels, the BFB group improved significantly (−16.8 mm), but the Control group (+0.5 mm) did not. A high (r = 0.83, p < 0.01) correlation could be found between the HHD Test and passive knee extension improvement after 6 weeks.

The passive knee flexion deficit of both groups was preoperatively similar (mean 8.1° ±8.7°). Flexion deficit of 68% of all patients was preoperative 0-5°. Both the EMG BFB group (<40°) and Control group (<40°) showed their largest deficits after 1 week. After 6 weeks the BFB group (20°) had a reduced, but not significantly different, flexion deficit compared to the Control group (26.3°).

### Subjective functional evaluation

The results of standard functional scoring scales (IKDC guidelines; [20,31] showed no significant differences between the groups during the first 6 weeks of rehabilitation. Both groups started preoperatively between “nearly normally” and “abnormally” knee joint function. After 1 week postoperative, both groups showed the strongest restriction (EMG BFB group 3.3 ± 0.7 / Control group 3.3 ± 0.9) and both reached a “nearly normally” knee function after 6 weeks (EMG BFB group 2.0 ± 0 / Control group 2.1 ± 0.6). The parameter “Activity of daily life” showed similar results. After 6 weeks of rehabilitation, both groups reported between “no” and “a bit” of ADL restriction. It seems that EMG BFB therapy had no influence on “joint function” (p = 0.72) and “ADL” (p = 0.96).

**Figure 7:**
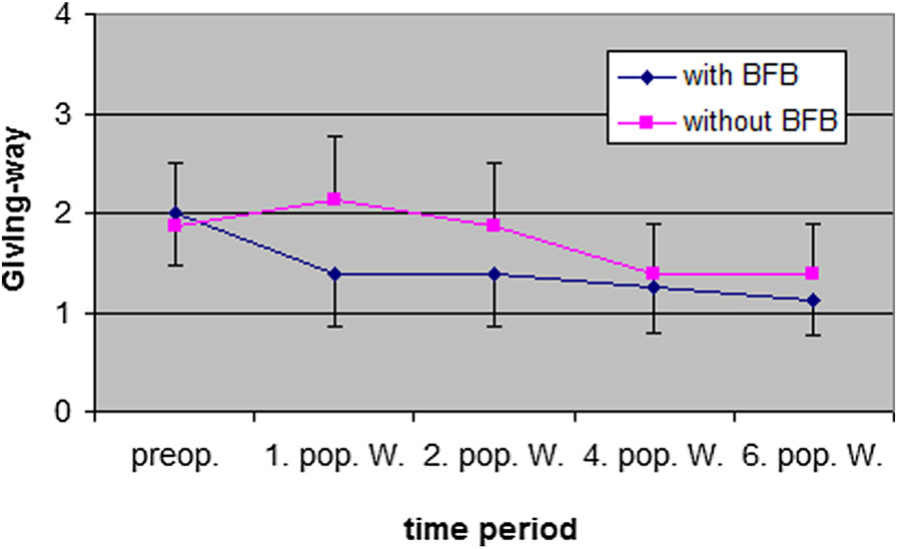
“Symptomatic giving way ” patients (mean value ± SD) of the affected knee preoperative and 6 weeks follow up (4 = Always, 3 = Frequent, 2 = Partly, 1 = None / preop. = preoperative, 1., 2., 4., and 6. Post-op. / W. = 1., 2., 4., and 6. Post-operative week).

The term “ Symptomatic giving way” describes the neuromuscular deficits of the quadriceps muscle, in the form of abrupt loss of tension during walking in the early phase of rehabilitation. The results (Figure 7) show differences between the two groups in the first and second postoperative week. After 6 weeks both groups improved their preoperative level (EMG BFB group 1.1 ± 0.4 / Control group 1.4 ± 0.5). The statistical analysis reported no significant differences between the two groups.

### Symptoms

Mean values and standard deviation of the visual analog scale (VAS 0–10) and knee swelling (categories 1 = none, – 4 = severe) also showed no significant differences between the two groups after 6 weeks rehabilitation. All patients reported their main pain under the patella, in the knee hollow, or in the wound area where the graft was taken. After 6 weeks the pain value of both groups was nearly “1”. The main knee swelling was located above the patella and at the medial joint gap. The strongest swelling was after 1 week (EMG BFB group 3.3 ± 0.5 / Control group 3.3 ± 0.9).

### Integrated EMG (vastus medialis)

**Figure 8:**
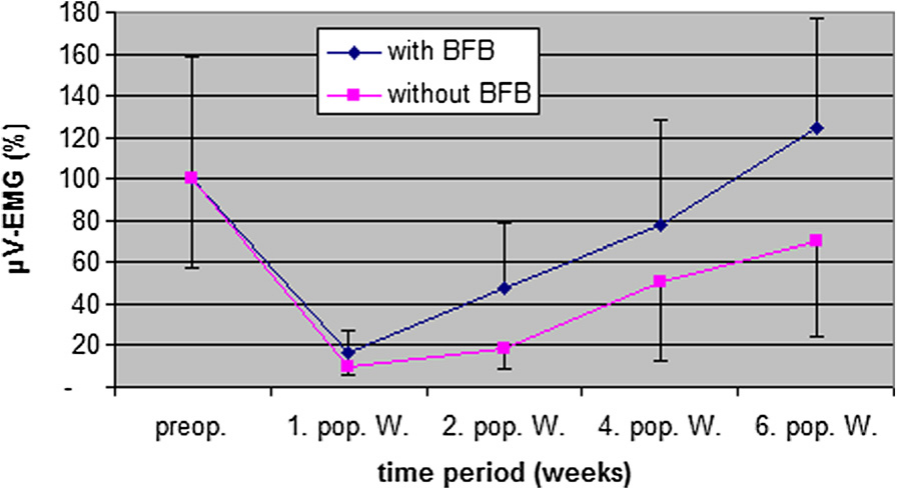
Comparison of percentage vastus medialis contraction (% μV-EMG) within two groups (mean value ± SD) pre- and postoperative 6 weeks follow up (preop. = preoperative, 1., 2., 4., and 6. pop. W. = 1., 2., 4., and 6. postoperative week).

Figure 8 shows the mean values with SD of percentage isometric vastus medialis contraction within the two groups during the 6 weeks rehabilitation period. Because of different inter-individual EMG values, and for better comparison, we took the preoperative value as 100%. Both groups showed the lowest percentage of EMG after the first postoperative week (EMG BFB group 16.7% ± 10.3% / Control group 9.7% ± 4.2%). During the 6 weeks rehabilitation period, the BFB group improved their EMG values significantly more (p < 0.01) than the Control group, so that after 6 weeks the BFB group (124.9% ± 52%) exceeded their preoperative level by 24.9%, while the Control group (70.3% ± 45.8%) deteriorated by 29.7%. A moderate correlation could be found between EMG and HHD Test improvements (r = −0.60 = p < 0.01) and between EMG and passive knee extension improvements (r = −0.71 = p < 0.002) after 6 weeks.

## Discussion

The deficit in knee extension (measured with goniometer) (Figure 5) showed a significant positive impact from BFB therapy on passive knee extension after 6 weeks of rehabilitation (p < 0.002). This result was corroborated by the HHD Test (Figure 6), which also reported significant differences after 6 weeks (p < 0.01), thus confirming the positive effect of BFB therapy on knee extension. Due to the significant reduction in the loss of knee extension, the concerns associate with premature muscle-strengthening training [19] and increased activities of ASMA (actin isoform alpha-smooth muscle actin) in the wound area [8] through the use of BFB therapy, can be rejected. However, our experience gained during therapy confirmed that wound healing periods, especially the first 3 postoperative weeks (phase of proliferation), should be respected. A high correlation between the HHD Test and knee extension (measured with goniometer) after 6 weeks (0.83 = p < 0.01) indicates that these tests are important and efficient instruments for use in the evaluation of ACL rehabilitation. EMG BFB therapy could help patients be more aware of correct muscle activity. The performance assessment may be an important source of motivation for patients. The results indicate that the application of EMG BFB therapy, in the early phase of rehabilitation, is useful in enhancing knee extension after ACL reconstruction.

Questions on the functional evaluation (joint function, ADL and “Giving way”) of the involved knee were asked 1 week before surgery and at periods of 1, 2, 4 and 6 weeks after reconstruction. The division of such parameters were based on IKDC categories, which are commonly used for the evaluation of ACL rehabilitation [14,24]. The results indicated that BFB therapy had no significant effects on joint function (p = 0.721) and ADLs (p = 0.959) compared to controls after 6 weeks rehabilitation. Our study does not show whether these findings could be different after a longer follow up period. The outcomes indicated that these parameters are not useful in evaluating knee function during the early phase of rehabilitation. Using a longer follow up of 12 months, Mihaela [26] showed a similar improvement in her study by using the “Knee Injury and Osteoarthritis Outcome Score” (KOOS).

BFB therapy showed positive effects on “Symptomatic giving way” (Figure 7) after 1 and 2 weeks (p = 0.028). This effect could be explained by the reduction of neuromuscular quadriceps deficit with BFB therapy, described by Krogsgaard & Solomonow [32] and Kanemura [33]. The application of BFB therapy could have positive influences on reduced afferent inputs (ligament, muscle, tendon, skin) as a protective function created by the central neural system. In addition, the results raised the question about the time period for using a brace. After 4 weeks, both groups showed almost no “Symptomatic giving way”. At this moment, most patients described wearing a brace as a restriction to the development of physiologic gait. This finding leads to a consideration of whether the recommended time period for wearing a brace should be less than 6 weeks.

Symptoms (knee pain – VAS; knee swelling) of the involved knee were measured 1 week before surgery and at the 1,2,4 and 6-week post-operative follow-ups. Neither the results of knee pain, nor of knee swelling, showed a positive effect from EMG BFB therapy after 6 weeks of rehabilitation, although many authors [34-36] confirm the relationship between knee pain, quadriceps inhibition and loss of knee extension. Concerning symptoms, it remains unclear whether BFB therapy could have an influence on later rehabilitation development. Muller et al. [37] investigated the wound area of Ligamentum patellae after 4.3 years (where the autograft was taken) and discovered that there was some coherence between knee pain and sub-optimal rehabilitation (shortening of Ligamentum patellae, increased quadriceps inhibition). The results of flexion deficit are of interest. The BFB therapy showed a positive, but not significant, influence on flexion deficit after both 2 (p = 0.05) and 6 (p = 0.05) weeks postoperatively.

This positive effect of BFB therapy, without additional knee flexion techniques, leads to the conclusion that therapy content for improving flexion could place more emphasis on reinforced strength and/or proprioceptive sessions during BFB therapy.

The literature has shown many EMG measurements, which were used as diagnostic [36,38-41] and/or therapy methods [25,31,42,43]. Selection of the vastus medialis muscle was based on Jarvela [44], who reported that the inferior fibres of vastus medialis are the most important for dynamic knee stability and the reduced load of passive knee structures. Because of different inter-individual EMG values (μV), to gain a better comparison the preoperative values were calculated relatively as 100%. The subsequent measurements at 1, 2, 4 and 6 weeks after ACL reconstruction were compared with this preoperative starting level.

The results of Figure 8 showed the biggest μV reduction 1 week postoperative (BFB Group 16.7%; Control group 9.7%). These findings are similar to other results, which also describe a protection mechanism by the central neural system in the form of quadriceps inhibition after ACL reconstruction [8,11,20,21] . Following the first postoperative week, the BFB Group improved their EMG values compared to the Control group, so that after 6 weeks the BFB Group (124.9% ± 52%) raised their preoperative level by +24.9%, while the Control group (70.3% ± 45.8%) deteriorated by −29.7%.

These results indicated the positive influence of BFB therapy on vastus medialis activation after both 2 (p < 0.01) and 6 weeks (p < 0.01). Recent studies have reported a long term effect of this training [5,45]. In addition, significant correlation between EMG and HHD Test (−0.602 = p < 0.01), and EMG and passive knee extension (−0.712 = p < 0.002), confirmed the relationship between vastus medialis innervation and knee extension. It is still unknown, whether there is a relationship between increased EMG values and improved quadriceps strength. Onishi [46], for example, found a correlation coefficient of 0.87 – 0.94 between EMG and strength.

Although it should be clear that EMG values are dependent on compliance and daily constitution of patients, and of innervation zones under attached electrodes., The fact is, that many authors [5,12,40,44,45,47] have described the persistent weakness of quadriceps as one of the biggest problems of ACL rehabilitation. In addition, they are convinced that reduced quadriceps strength can be traced back to sub-optimal rehabilitation, due to a persistent spinal or supraspinal reflex inhibition. It may be that improved activation of the vastus medialis through EMG BFB therapy could lead to a subsequent support for improved quadriceps strength. It remains unknown whether EMG BFB therapy in the early phase of rehabilitation could have a positive influence on subsequent increased knee-loading, on return to sporting activities. Recent studies conclude that an early functional EMG BFB therapy can have a positive effect for a better return to sport activities [5,45].

### Limitations

The sample size of 16 subjects was small. The patients and the assessor were not blinded. The treating therapist also performed the measurements and may have introduced bias. Future studies should include larger samples, blinded assessors and longer follow up times.

## Conclusion

These results indicate that EMG BFB therapy, in the early phase of rehabilitation, is useful in enhancing knee extension after ACL reconstruction. Improved innervation of the vastus medialis seems to play a key role in the development of postoperative knee extension. EMG BFB is a simple, inexpensive and valuable adjunct to conventional therapeutic modalities.

## Competing interests

The authors declare that they have no competing interests.

## Authors’ contributions

FCh planned the study, treated the patients, was involved in assessments, and was the main author of the paper. HC, HR, FC were involved in planning and executing the study, helped in statistical calculations, methodology and helped in the writing of the paper. HL was involved in the methodology of the study, proof reading of the paper, proving the statistics, graphical representation, and helped in writing the final version of the paper. All authors approved the final version of the paper.
